# Pulsatile arterial compression of cranial nerves

**DOI:** 10.4103/2152-7806.66458

**Published:** 2010-07-16

**Authors:** Joyce S. Nicholas, Sunil J. Patel

**Affiliations:** 1 Department of Medicine, Division of Biostatistics and Epidemiology, South Carolina, USA; 2 Department of Neurosciences, Division of Neurosciences, Division of Neurosurgery, Medical University of South Carolina, Charleston, South Carolina, USA

The authors are to be commended for their observations on patients undergoing retromastoid craniectomy with microvascular decompression of the right ventrolateral medulla (VLM) and vagus nerve for type 2 diabetes mellitus. Seven of the 10 patients observed demonstrated overall glycemic control that either improved or did not worsen at the 12-month postoperative follow-up visit.

These observations point the way to further questions that need to be answered to conclude definitively that pulsatile arterial compression of the right VLM is an independent risk factor for type 2 diabetes mellitus. As stated by the authors, a neurogenic basis for various cranial nerve hyperactive syndromes has long been postulated in the literature, with the first on essential hypertension made by Dr. Jannetta in the 1970s. Animal studies confirmed the presence of a subpial catecholamine-synthesizing neuronal group (C1) in the rostral ventrolateral medulla, which produces a transient pressor response when stimulated electrically, chemically or mechanically.[3,4,7,10] Histochemical studies of human medullae obtained at autopsy showed a similar group of catecholamine neurons in the subpial regions of the retro-olivary sulcus (ROS) near the root-entry zone of the ninth and tenth cranial nerves.[1,6] Conflicting results from early studies of the association between arterial compression and essential hypertension may have been due in part to inconsistencies in the exact location of compression or the failure to identify the subgroup of hypertensive patients most likely to benefit from microvascular decompression. Later studies began to address these questions. It was hypothesized that arterial pulsatile compression may either directly stimulate the C1 neurons or disinhibit them via an effect on the caudal ventrolateral medulla resulting in elevation of central sympathetic outflow or tone.[5] In support of this hypothesis, it was found that in a group of 18 subjects, those with vascular contact along the left ROS (presumed location of the C1 neurons) had higher baseline plasma norepinephrine concentrations and greater clonidine depressor response (sympathetic dependence of blood pressure) than those without vascular contact along the left ROS.[5] To better address the question of location, electrical stimulation studies were undertaken to precisely define the location of the sympatho-excitatory and inhibitory neuronal aggregates in humans.[9,11] A defined area of hypertensive responses was found at the mid ROS, centered 6 mm rostral to the lowest nerve root. Microneurography data suggested that the observed elevation of arterial blood pressure associated with electrical stimulation of this area was due to an activation of the sympathetic system, with no conclusive evidence of lateralization.[9,11] The question of whether arterial compression acted as an independent predictor of essential hypertension (apart from other known risk factors) was addressed in larger multivariable retrospective designs.[2,8] In a case-control study of 147 patients, it was found that the odds of arterial compression among hypertensive subjects were 2.99 times the odds among normotensive subjects (*P*= .04), controlling for hypertension risk factors such as age, body mass index, race, diabetes and family history of hypertension. Of compressed hypertensive subjects, 56% were compressed on the left and 44% on the right.[8] Collectively, these studies are building a body of evidence to aid the selection of patients eligible for microvascular decompression, i.e., those with intractable hypertension, elevated sympathetic tone, and arterial compression in accordance with a refined map of the human ventrolateral medullary surface and its relationship to cardiovascular control.

Like Dr. Jannetta’s earlier observations on essential hypertension, the observations presented in the manuscript “Type 2 Diabetes Mellitus: A Central Nervous System Etiology” are valuable starting points for questions related to the exact location of arterial compression relevant to type 2 diabetes mellitus, the best experimental measure of response, and the subset of patients most likely to benefit from microvascular decompressive surgery. The authors have made a start in this direction by observing that non-obese patients with type 2 diabetes mellitus appear to respond better to decompression than obese patients. We encourage their continued efforts and those of other researchers in addressing those questions raised by this valuable contribution to our understanding of type 2 diabetes mellitus and its treatment.
